# Immunomodulatory Effect of *Helleborus purpurascens* Waldst. & Kit.

**DOI:** 10.3390/plants10101990

**Published:** 2021-09-23

**Authors:** Alice Grigore, Corina Bubueanu, Lucia Pirvu, Georgeta Neagu, Ionica Bejanaru, Virginia Vulturescu, Minerva Panteli, Iuksel Rasit

**Affiliations:** National Institute for Chemical-Pharmaceutical Research and Development—ICCF Bucharest, Calea Vitan No. 112, 3rd District, 031299 Bucharest, Romania; corina.bubueanu@yahoo.com (C.B.); lucia.pirvu@yahoo.com (L.P.); ionicabejanaru@yahoo.com (I.B.); virginiavulturescu@yahoo.com (V.V.); minerva.panteli@gmail.com (M.P.); iuksel_rasit@yahoo.com (I.R.)

**Keywords:** hellebore, *Ranunculaceae*, ecdysones, immunostimulation, caffeic acid

## Abstract

(1) Background: *Helleborus purpurascens* Waldst. & Kit. (hellebore) is a plant species found mainly in Balkans and the Carpathians, and it is traditionally used for a variety of ailments since the time of Hippocrates. The aim of this study was to investigate the immunomodulatory effect of hellebore extracts correlated with relevant chemical compounds and the extraction method. (2) Methods: A methanolic (H1) and a hydroalcoholic extract (H2) were prepared by standard methods. Qualitative (HPTLC) and quantitative (HPLC) chemical analysis were conducted to reveal the ecdysones and polyphenolic compounds. In vitro studies were performed using rat macrophages, murine fibroblasts and immortalized human T-lymphocytes, and their viability was determined by MTS assay. In vivo studies involved a rat immunodepression model. (3) Results: In vitro assays revealed the stronger effect of H2 on cellular proliferation, compared to H1. In the in vivo assay, H2 revealed an immunostimulatory effect in the context of experimentally induced immunosuppression with dexamethasone, a superior effect to levamisole treatment according to the same regimen, in two doses every 24 h. There was no correlation between pharmacological effect and the reference compounds evaluated. (4) Conclusions: The immunomodulatory effect of methanolic and hydroalcoholic hellebore extracts is not due to ecdysones and polyphenolic compounds, but other polar substances, possible steroid glycosides.

## 1. Introduction

From a botanical point of view, *Helleborus purpurascens* Waldst. & Kit. (hellebore) belongs to the Ranunculaceae family, being one of the 20 species of the genus *Helleborus*. The highest concentration of species is found in the Balkans and the Carpathians, and it is traditionally used for a variety of ailments since the time of Hippocrates.

Studies have been performed to evaluate the therapeutic and pharmacological effects of raw or selective extracts of some *Helleborus* species using various animal models or in vitro systems [1]. Kerek (2005) [2] presented for the first time the beneficial action of the drug Boicil prepared from the root extract and stems of *H. purpurascens* used successfully in Romania for the treatment of rheumatic pains. *H. purpurascens* extracts exert a long-lasting analgesic and muscle relaxant action. The immunostimulatory effect of *H. purpurascens* has been reported in sheep; the increase in the number of lymphocytes (2×) and neutrophils (3.5×) 48 h after the injection of 5% decoction of roots and rhizomes showed the improvement of the immune response in these animals [3]. Rapid and nonspecific activation of defense mechanisms (pronounced leukocytosis and increased neutrophil percentage) has been reported after subcutaneous, intraperitoneal and intramuscular administration of different concentrations of *H. odorus* extract [4]. The immunomodulatory action has been attributed to a compound called MCS-18 (macrocyclic carbon suboxide), a multi-anionic compound extracted and purified from the roots of *H. purpurascens* and classified as new chemical entity (NCE) 88, which has been characterized as an inhibitor of Na and adenosine triphosphatases (K-ATPases and SR Ca-ATPases) [5]. MCS-18 has been extensively studied; it upregulates the response of the immunomodulatory cytokines IL-10 and TGF-β [6] and the formation of T-cell-dependent antibodies in mice likely by blocking Toll-like receptors. MCS-18 is also an antagonist of capsaicin-activated vanilloid pain receptors (TRPV1), which explains the analgesic action [7]. Studies have shown that MCS-18 exerts an enhanced immunosuppressive action with remarkable therapeutic potential in the therapy of diseases characterized by a pathological hyperactivation of the immune response. For instance, it was showed that in atherosclerotic lesions, MCS-18 suppresses the recruitment of immune cells into the inflammatory site, with focus mainly on dendritic cells, which are associated with plaque vulnerability [8]. Thionines—a class of peptides found in *H. purpurascens*—have been proposed as potential immunotoxins in tumor therapy. The anticancer properties of thionines have been reported by Kerek [9]. By acting synergistically or singularly, helethionines inhibit the proliferation of tumor cell lines. For example, helethionine C (2 µg/mL) in combination with MCS-18 inhibits the proliferation of the MFC-7 line (breast cancer line). Helethionines C and D, at a concentration of 100/g/mL, inhibit (48.36% and 58.66%, respectively) the Colo 205 culture (colon cancer line). A mixture of helethionine B (B1/B2/B3 = 1:1:1), helethionine C and D at a concentration of 100 µg/mL inhibits by 50% the growth of a line of lung carcinoma [9].

This paper focuses on the immunomodulatory effect of hellebore extracts correlated with relevant chemical compounds and the extraction method. Moreover, considering the ecdysteroids as one of the representative classes in the *Helleborus* genus, we highlight their possible involvement in immunomodulation. The *Helleborus* genus is a species ethnopharmacologically used since ancient times in Southeast Europe. Although it contains toxic substances, its medicinal benefits are important and recognized, and research on its chemical composition and pharmacology is imperative. Our study brings new information about a scarcely studied plant species, focusing also on safety and efficacy aspects of introducing a medicinal natural product in medicinal practice.

## 2. Results and Discussion

### 2.1. Chemical Analysis

**Figure 1:**
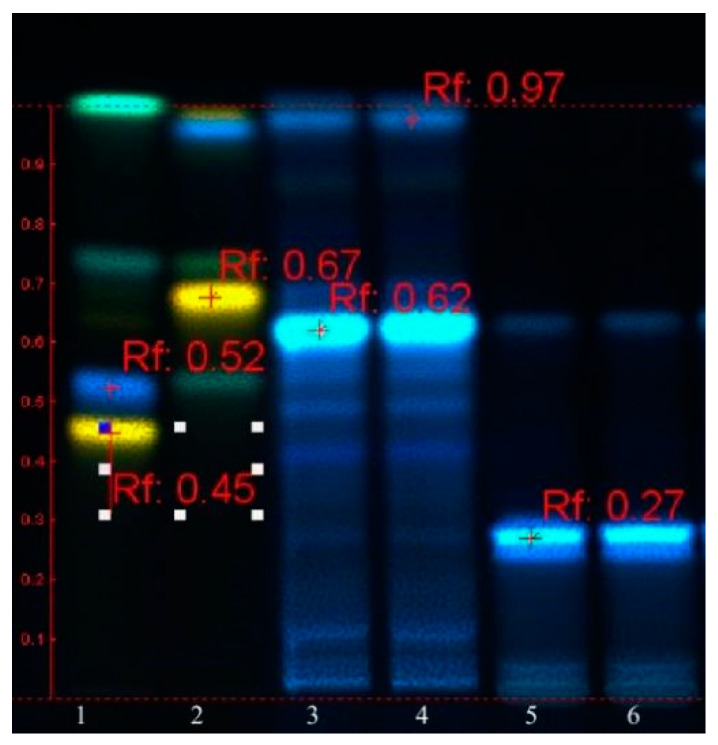
HPTLC fingerprint of polyphenols in *Helleborus* samples. Track 1: reference substances (*Rf* ≈ 0.45—rutin, *Rf* ≈ 0.52—chlorogenic acid); track 2: *Rf* ≈ 0.67—hyperoside; tracks 3 and 4: H1 (duplicate); tracks 5 and 6: H2 (duplicate).

HPTLC analysis confirmed the presence of polyphenols and ecdysones in both samples. The absence of flavones and also the presence of caffeic acid Rf ~0.97 as well as other blue spots at Rf ~0.27, 0.62, 0.89 are noticed (Figure 1).

**Figure 2:**
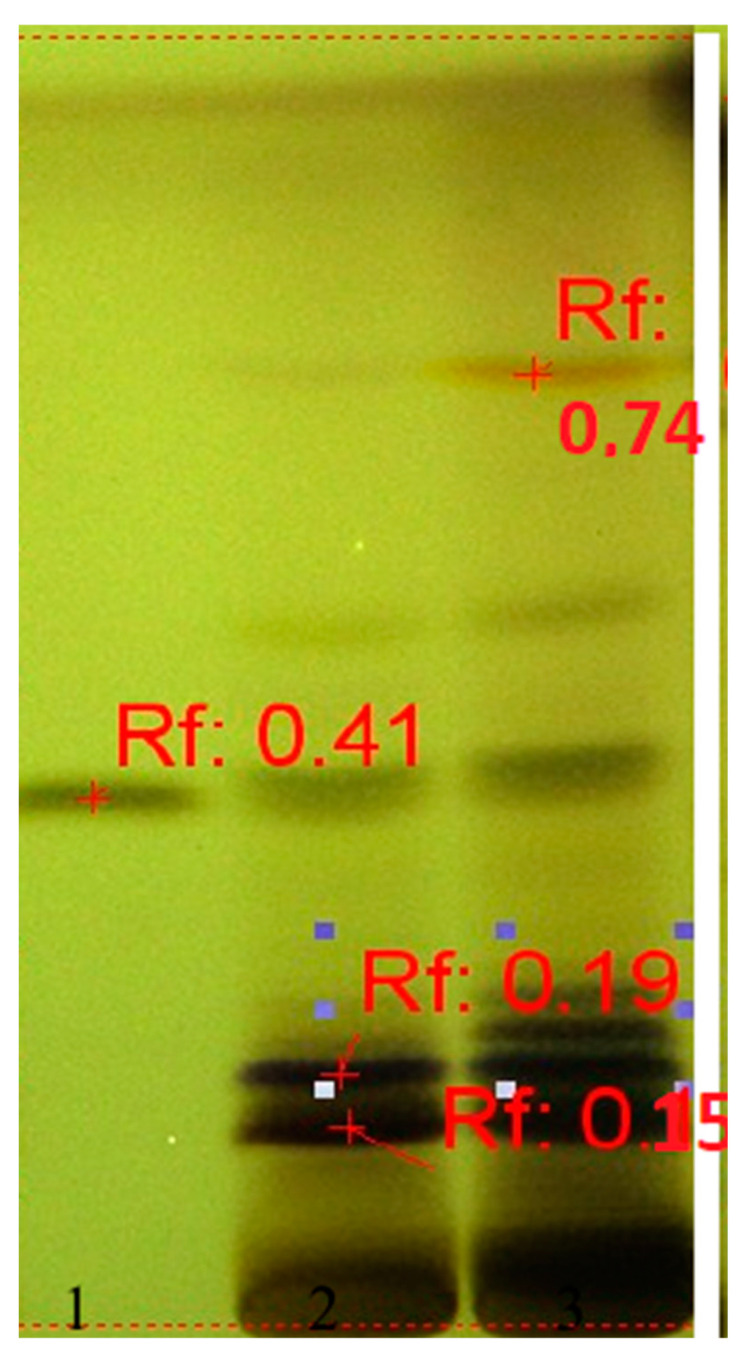
HPTLC fingerprint of ecdysones in *Helleborus* samples. Track 1: reference substance β-ecdysone, *Rf* ≈ 0.41; track 2: H1; track 3: H2.

The presence of β-ecdysone in both extracts was highlighted at Rf ~ 0.41 and that of the cardiac glycosides of the hellebrin type at Rf ~0.15 and 0.19 (Figure 2), identified according to Plant Drug Analysis guidelines [10].

The two *Helleborus* samples were standardized in caffeic acid and β-ecdysone. It is well known that, chemotaxonomically, the *Helleborus* genus is characterized by the presence of steroids, including bufadienolides, phytoecdysones and steroidal saponins [11]. HPLC analysis (Figures S1–S4) revealed that the H1 sample obtained by maceration of the raw material with methanol is richer in selected compounds compared to the H2 sample (Table 1). 

### 2.2. In Vitro Assays

We investigated the viability of several cell lines treated with increasing concentrations of *H. purpurascens* extracts (9.37–500 μg/mL) for 24 and 48 h using the MTS assay, as described in Materials and Methods.

Both extracts exhibited a dose-dependent inhibitory effect on all cell lines. The IC_50_ of H1 was 54.71 μg/mL against L929 cells and 20.27 μg/mL against NR8383 macrophages, while the IC_50_ of H2 was twice as high—95.3 μg/mL against L929 cells and 40.28 μg/mL against NR8383 macrophages.

**Figure 3:**
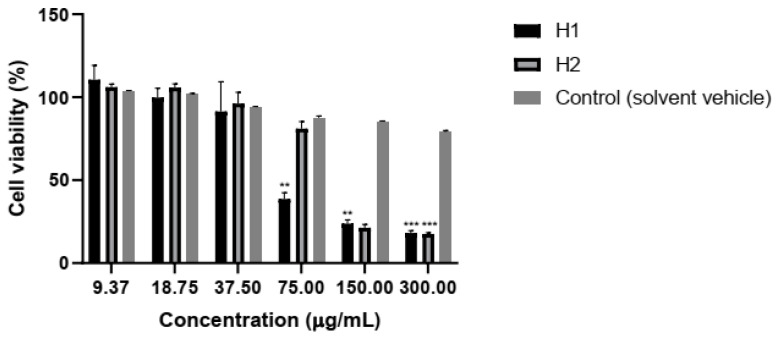
Viability of L929 cells after 24 h exposure to hellebore extracts. Values represent mean ± SD (n = 3, ** *p* < 0.01, *** *p* < 0.001).

In L929 cells, the viability drops under 50% after administration of H1 at doses of 75–300 μg/mL, while H2 is toxic only at the highest doses tested (Figure 3). 

**Figure 4:**
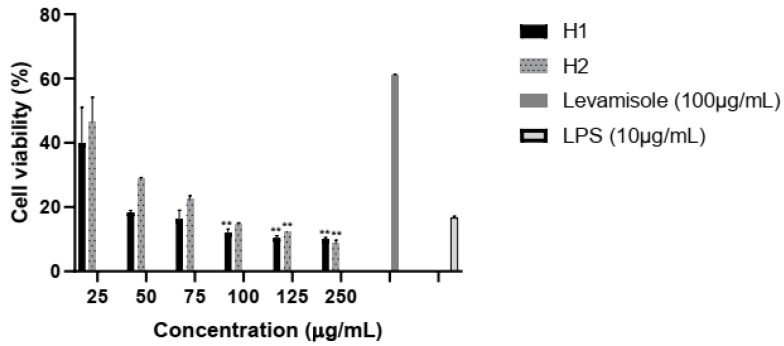
Viability of NR8383 cells after 18 h exposure to hellebore extracts, and LPS (10 μg/mL). Levamisole (100 μg/mL) was used as positive control and LPS alone (10 μg/mL) as negative control. Values represent mean ± SD (n = 3, ** *p* < 0.01).

In murine macrophages, LPS alone exhibits a strong inhibitory effect after 21 h exposure. By adding doses of 25–100 μg/mL of H2, cells viability increases up to 50% showing the synergism of herbal extract with LPS for macrophage activation (Figure 4). 

**Figure 5:**
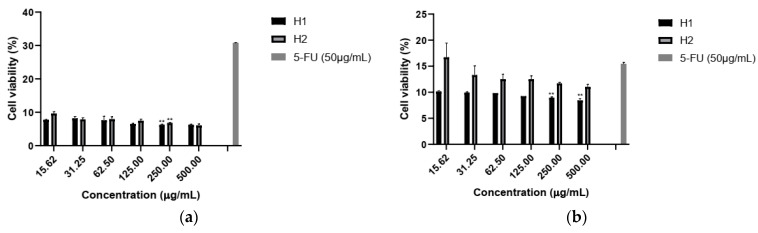
Viability of Jurkat cells after (**a**) 24 h and (**b**) 48 h exposure to hellebore extracts. Positive control, 5-fluorouracil (5-FU) (50 μg/mL). Values represent mean ± SD (n = 3, ** *p* < 0.01).

Jurkat cells were strongly inhibited by both extracts at all concentrations, after 24 and 48 h exposure. H1 was more efficient; the viability after 48 h remained under 10% (Figure 5). 

In general, in vitro assays revealed the stronger effect of H2 on cellular proliferation, compared to H1. Reference compounds used to standardize the extracts—caffeic acid and ecdysone—are well documented for their antitumor or immunomodulatory effects [12,13]. 20-hydroxyecdysone isolated from the butanolic extract of the underground parts of *H. caucasicus* exerts a strong cytotoxic effect on the cancer cell line of lung origin (Calu-1) and on other cancer cell lines (HepG2 and Caco-2), reducing the S-phase entry and inducing cell apoptosis [14]. 

The pharmacological effect of both extracts but mainly H1 on Jurkat cells could be attributed to another characteristic class of compounds for the genus *Helleborus*, ecdysteroids, which are easily extracted by methanol. The cytotoxic potential of these compounds is very well documented. Steroidal compounds thibetanoside C isolated from *H. thibetanus* showed cytotoxicity against A549 cells (IC_50_ 39.6 ± 1.9 μM) and HepG2 cells (IC_50_ 41.5 ± 1.1 μM), respectively, and 23*S*, 24*S*)-24-[(*O*-β-D-fucopyranosyl)oxy]-3β, 23-dihydroxy-spirosta-5, 25(27)-diene-1β-yl*O*-(4-*O*-acetyl- α-L-rhamnopyranosyl)-(1→2)-*O*-[β-D-xylopyranosyl-(1→3)]-α-L-arabinopyranoside showed cytotoxicity against HCT116 cells (IC_50_ 33.6 ± 2.1 μM) [15]. The possible mechanism involves caspase-9-dependent apoptosis, double strand DNA breaks, Jun N-terminal kinases (JNK) activation and suppression of signal transduction pathways that control key cellular processes such as transcription, translation, cell cycle progression and apoptosis [16].

Bufadienolides from *H. foetidus* displayed potent cytotoxicity against HL-60 and A549 cells, with IC_50_ values of 0.035 and 0.029 μM, respectively [17].

Bufadienolides from *H. lividus* showed cytotoxicity against HL-60 human leukemia cells and A549 human lung adenocarcinoma cells, with IC_50_ values ranging from 2.20 ± 0.01 nM to 0.77 ± 0.01 μM via a mechanism of action other than Na^+^/K^+^ ATPase inhibition [18].

### 2.3. In Vivo Assay

For investigation of hellebore immunostimulatory potential, we conducted a classic assay using dexamethasone as immunosuppressive agent and levamisole as positive control, known to induce a strong cellular immune response.

There were no clinical signs of toxicity or mortality in animals treated with dexamethasone and those treated with levamisole, H1 or H2.

Laboratory investigations were performed on the hematological parameters presented by the mean values ± standard deviation (Table 2). The hematological parameters followed were WBC = leukocyte count; LYM = lymphocyte count, GRA = neutrophil count, LY% = lymphocyte%, GR% = neutrophil count%, RBC = erythrocyte count, Hb = hemoglobin, HCT = hematocrit, PLT = platelet count, MCV = mean corpuscular volume, MCH = hemoglobin mean erythrocyte, MCHC = mean hemoglobin concentration. The tests were performed using the Abacus Junior Vet automatic hematology analyzer.

The total number of leukocytes and the leukocyte formula are the main hematological indicators of immune status. The total number of leukocytes on the seventh day of the experiment is as follows: Group V > Group I > Group III > Group IV > Group II. The increased value of leukocytes is recorded in diseases that require the body’s cellular defense reactions. Thus, the low value in Group II expresses an immunodeficiency installed with susceptibility to infections.

Neutrophil/lymphocyte ratio was used in assessing immune and inflammatory status. The values of the neutrophil/lymphocyte ratio are: Group I (0.57), Group II (0.85), Group III (0.82), Group IV (1.31), Group V (3.06). The lowest value of the neutrophil/lymphocyte ratio between the treated groups appears in Group II, a sign of the immunosuppressive action of dexamethasone. The groups treated with H2 and H1 showed values higher than the group treated exclusively with dexamethasone, a sign of immune stimulation in the context of induced immunosuppression. Moreover, both extracts induced a marked increase of granulocytes, of 25% for H1 and 51% for H2 compared to Group II treated only with dexamethasone. Granulocytes are the first base in initiation of infectious or inflammatory processes and their rapid stimulation proves that hellebore extracts exhibit a role in the activation of the innate immune response.

The number of erythrocytes on the seventh day of the experiment is as follows: Group V > Group II > Group III > Group IV > Group I. The increased value obtained for Group V suggests a possible effect of stimulating the erythrocyte line of the H2 sample.

From the table of the values of the body weight of the animals from the experiment during the 6 days (Table 3), the following results can be observed: The first control group evolves upwards during the monitoring period; in groups II, III, IV and V there is a marked weight loss since the beginning of dexamethasone treatment, with a tendency to return after the second intake of levamisole/H1/H2.

Given that H2 extract is more potent in inducing an immunostimulatory effect, it could be hypothesized that polar compounds are responsible for the pharmacological effect. Even if H1 extract is richer in marker compounds—ecdysone and caffeic acid—it fails to induce a strong response compared to the H2 sample.

## 3. Materials and Methods

### 3.1. Plant Material

*Helleborus purpurascens* Waldst. & Kit. was cultivated under ecological conditions by S.C. Dacia Plant S.R.L., Bod, Romania, and dried and ground as a fine powder. A voucher specimen (HP124/2019) was deposited at the manufacturer.

### 3.2. Preparation of the Hellebore Extracts

The first extract (H1) was prepared by soaking 150 g of Hellebori radix in 1500 mL methanol (1:10 *w/v*) for 2 days at room temperature, followed by filtration. The crude extract was concentrated under reduced pressure (72–74 mmHg), and the resulting *Extractum spissum* (27.56 g) was dissolved in 20% ethanol to obtain a stock solution of 250 mg/mL. The second sample (H2) was prepared by extracting 150 g of Hellebori radix in 1500 mL ethanol 30% (1:10 *w/v*) under reflux for 30 min, followed by filtration. The crude extract was concentrated under reduced pressure (72–74 mmHg), and the resulting *Extractum spissum* (38.18 g) was dissolved in 20% ethanol to obtain a stock solution of 250 mg/mL.

### 3.3. HPTLC Analysis

Chromatography was performed on silica gel F254 HPTLC pre-coated plates using as mobile phase ethyl acetate–formic acid–glacial acetic acid–water (100:1:11:26) for identification of polyphenols [10] and ethyl acetate–methanol–water (81:11:8) for ecdysteroids [10]. Samples were applied on the plates as band of 7 mm width using a Camag Linomat V sample applicator at the distance of 14 mm from the edge of the plates. The fingerprints were evaluated in visible mode with a WinCats software after derivatization with NP-PEG for polyphenols or after drying the plate at 110 °C for 10 min, for ecdysone visualization. Reference compounds for HPTLC analysis were purchased from Sigma-Aldrich, Germany (rutin, chlorogenic acid, hyperoside) and from PhytoLab, Baden-Wurttemberg, Germany (β-ecdysone).

### 3.4. HPLC Analysis

Quantitative HPLC analysis of main components was performed on a HPLC ELITE—LaChrom system, with a DAD detector and a Luna C18(2) column (250 × 4.6 mm, 5 μm) at 23 °C. Separation of polyphenols was performed using a mobile phase consisting of an A solution (water acidified with phosphoric acid, pH = 3.0) and a B solution (acetonitrile), under a gradient program with an initial flow rate of 1 mL/min: 0 min: 90% A-10% B; 30 min 10% A-90% B; 31 min 0% A-100% B; 35–55 min 90% A-10% B. For determination of ecdysone content, the abovementioned column was used along with a mobile phase consisting of 20% aqueous acetonitrile for isocratic elution at an initial flow rate of 1.5 mL/min with an injection of 20 μL. Reference substances were purchased from Sigma-Aldrich, Germany (caffeic acid) and from PhytoLab, Baden-Wurttemberg, Germany (β-ecdysone).

### 3.5. In Vitro Tests

The assay was used to assess the inhibitory effects of the extracts on the survival of several types of cell lines—mouse fibroblasts (L929), rat alveolar macrophages (NR8383), immortalized human T-lymphocytes (Jurkat). A total of 1 × 10^3^ (L929 and NR8383) and 2.5 × 10^3^ (Jurkat) cells/well were seeded into 96-well plates and were allowed to settle down for 24 h. The cells were then treated with several dilutions of extracts. Each concentration/assay was performed three times. The 20% ethanol (extracts solvent) was diluted with culture media in the same way as samples and served as a negative control. Levamisole (100 µg/mL) and 5-fluorouracil (5-FU) (50 µg/mL) were also used as positive controls. The cells were incubated with the treatments for 24–48 h, after which 20 μL MTS (5 mg/mL, CellTiter-Aqueous One^®^, Promega) was added and further incubated for 4 h at 37 °C. The absorbance was measured at 492 nm with an LKB Chameleon microplate reader. Cell viability was expressed as a percentage of live treated cells compared with live control cells.

For evaluation of survival of LPS-stimulated macrophages, cells were treated with serum-free media supplemented with several dilutions of extracts (15.62–500 µg/mL) in the presence of LPS (10 μg/mL). LPS alone served as negative control. After 18 h incubation, the cultures were also subjected to MTS assay as described above.

### 3.6. In Vivo Assay of the Immunostimulatory Potential

An immunosuppressive rat model was used. The rat immunodepression model is a widely used model for the evaluation of substances with immunomodulatory potential in organisms with depressed/compromised immune system.

#### 3.6.1. Animals and Housing Conditions

Male Wistar rats were provided by the Cantacuzino Institute’s biobase and maintained in theNCPRI animal house, Department of Pharmacology, under controlled conditions at 27 °C with 12 h variation of light and dark period. Water and feed were provided ad libitum. Male rats (270–330 g weight) were acclimatized for 1 week prior to the experiment.

Immunodepression was induced in rats using dexamethasone (5 mg/kg body weight (kg bw) intraperitoneally, in two daily doses for 3 days). The animals were observed twice daily for clinical signs and mortality, and body weight.

Drug treatment and experimental groups: After acclimation, 40 Wistar rats were divided into five experimental groups equally as follows: Group I—C—untreated white control—only food and water were administered, Group II—D—treated with dexamethasone 5 mg/kg bw intraperitoneally twice daily for three days, Group III—D + L—treated with dexamethasone 5 mg/kg bw intraperitoneally twice a day for three days followed by levamisole 7.5 mg/kg bw subcutaneously in two doses every 24 h, Group IV—D + H1—treated with dexamethasone 5 mg/kgb intraperitoneally twice a day for three days followed by H1 extract 12.5 mg/kg bw subcutaneously in two doses every 24 h, Group V—D + H2—treated with dexamethasone 5 mg/kg bw intraperitoneally twice a day for three days followed by H2 extract 12.5 mg/kg bw subcutaneously in two doses every 24 h.

All rats were sacrificed 24 h after the last dose administered, and blood samples were collected from the abdominal aorta.

#### 3.6.2. Ethical Statement

All animal studies were approved by the Ethical Committee of the National Institute of Chemical-Pharmaceutical Research and Development and carried out according to the National Medicine Agency regulations, Directive 2010/63/EU revising Directive 86/609/EEC on the protection of animals used for scientific purposes and Law no. 43/2014 on the protection of animals used for scientific purposes issued by the Romanian Parliament.

#### 3.6.3. Statistical Analysis

For the statistical analysis, paired Student’s t-test was used. The results were expressed as means ± SD of at least three independent experiments. *p* < 0.05 was considered to be statistically significant. All analysis were performed with GraphPad Prism (version 9.12; GraphPad Software Inc.; San Diego, CA, USA).

## 4. Conclusions

*Helleborus purpurascens* is a very valuable species, with high pharmacological potential. This study revealed that immunomodulatory effect of methanolic and hydroalcoholic hellebore extracts is not due to ecdysones and polyphenolic compounds but other polar substances, possible steroid glycosides. Further studies are needed to highlight the responsible compounds and the mechanism of action.

## Figures and Tables

**Table 1:** Reference compounds identified in hellebore extracts by HPLC.

Scheme 100	Reference Compound (mg/100 g Dry Extract)	
	β-Ecdysone	Caffeic Acid
H1	216.64 ± 0.5	56.48 ± 0.43
H2	143.16 ± 0.48	36.41 ± 0.35

**Table 2:** Hematological parameters (mean values ± standard deviation).

	Group I C	Group II D	Group III D + L	Group IV D + H1	Group V D + H2
WBC (×10^9^/L)	4.87 ± 1.254	2.77 ± 0.96	3.4 ± 0.81	2.82 ± 0.7	5.09 ± 2.85
LYM (×10^9^/L)	3.07 ± 0.944	1.37 ± 0.33	1.69 ± 0.74	1.11 ± 0.31	1.17 ± 0.27
GRA (×10^9^/L)	1.77 ± 0.695	1.16 ± 0.44	1.39 ± 0.31	1.46 ± 0.28	3.57 ± 2.84
LY%	63.3 ± 10.19	49.8 ± 4.16	48.8 ± 11.4	39.4 ± 2.48	30.7 ± 22.3
GR%	36 ± 10.1	41.8 ± 1.26	41.5 ± 10.4	52.4 ± 3.56	63.3 ± 18.6
RBC	8.8 ± 0.242	9.21 ± 0.41	8.87 ± 0.58	8.32 ± 1.3	9.29 ± 0.32
HGB	17 ± 0.058	17.6 ± 0.71	17.6 ± 0.67	17.5 ± 0.67	17.8 ± 0.26
HCT%	47.8 ± 1.518	50 ± 2.73	49.6 ± 2.76	49.5 ± 1.67	50.4 ± 0.91
MCV (fl)	54.3 ± 0.577	54.3 ± 1.15	56 ± 1.73	55 ± 1	54 ± 1
MCH (pg)	19.4 ± 0.513	19.1 ± 0.12	19.9 ± 1.02	19.5 ± 0.42	19.2 ± 0.4
MCHC (g/dL)	35.6 ± 1.054	35.3 ± 0.99	35.5 ± 0.95	35.3 ± 0.42	35.3 ± 0.1
PLT (×10^9^/L/L)	869 ± 169.1	611 ± 98.1	564 ± 116	693 ± 56,2	572 ± 74.1

**Table 3:** Evolution of the weight of the animals included in the experiment.

Days	1	2	3	4	5	6
weight	g	g	g	g	g	g
Group I	299.84	306.83	309.43	306.74	305.48	312.61
Group II	292.59	279.62	274.19	260.82	253.28	261.82
Group III	296.61	286.75	282.80	271.40	260.22	268.95
Group IV	303.89	295.36	282.82	270.97	266.94	276.17
Group V	297.22	292.58	277.90	264.62	256.06	258.32

## Data Availability

The data that support the findings of this study are available from the corresponding author upon reasonable request.

## Supplementary Materials

HPLC chromatograms are available online at www.mdpi.com/article/10.3390/plants10101990/s1.
