# Supplementary material for: Immunomodulatory Effect of Helleborus purpurascens Waldst. & Kit
Source: Plants (Basel). 2021 Sep 23;10(10):1990. doi: 10.3390/plants10101990 (PMC8538211; doi:10.3390/plants10101990)
Supplement: Supplementary file 1 [file plants-10-01990-s001.zip › plants-1364809-supplementary.pdf]

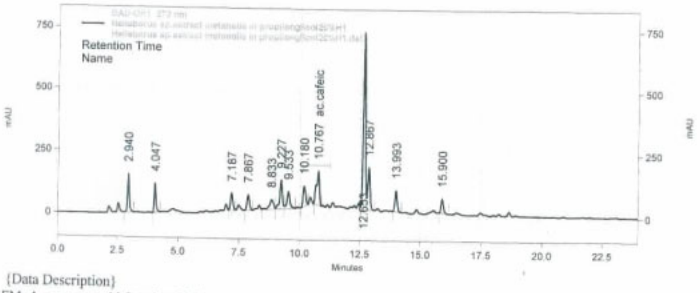

{Data Description}
FM. A = apa cu acid formic, pH 3
FM. B =ACN
Gradient
Temperatura =23 C
Debit = 1 ml/min
Detectie = 273 nm.
Coloana: Inertsil ODS 3 250*4.6*5 (42)

DAD-CH1 273 nm Results

Pk #	Name	RT	Area
9	ac.cafeic	10.767	4731011

DAD-CH1 273 nm Results

Pk #	Name	Height	IC	PLATES	K	Res	As	S/N
9	ac.cafeic	534888	BB	22580	6.2	2.4	0.8	8.7

Figure S1. HPLC chromatogram of H1 - caffeic acid

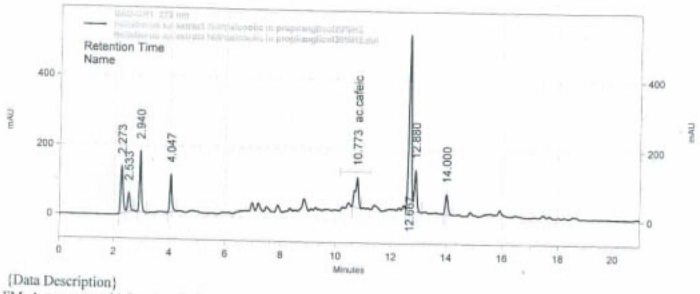

{Data Description}
FM. A = apa cu acid formic, pH 3
FM. B =ACN
Gradient
Temperatura =23 C
Debit = 1 ml/min
Detectie = 273 nm.
Coloana: Inertsil ODS 3 250*4.6*5 (42)

DAD-CH1 273 nm Results

Pk #	Name	RT	Area
5	ac.cafeic	10.773	3050335

DAD-CH1 273 nm Results

Pk #	Name	Height	IC	PLATES	K	Res	As	S/N
5	ac.cafeic	345214	BB	22740	6.2	32.5	0.8	4.0

Figure S2. HPLC chromatogram of H2 - caffeic acid

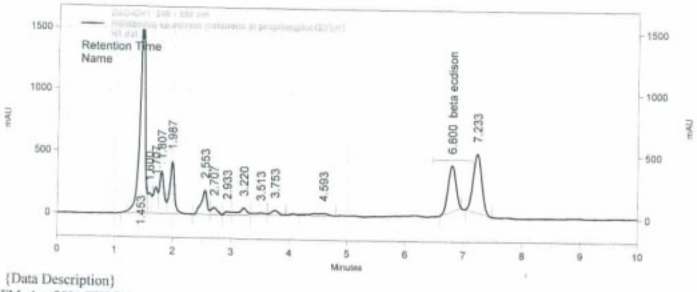

{Data Description}
FM. A = 20% CH3CN
Temperatura =55 C
Debit = 1,5 ml/min
Detectie = 248 nm.
Coloana: Inertsil ODS 3 250*4.6*5 (42)

DAD-CH1 248 - 350 nm Results

Pk #	Name	RT	Area
13	beta ecdison	6.800	13525081

DAD-CH1 248 - 350 nm Results

Pk #	Name	Height	IC	PLATES	K	Res	As	S/N
13	beta ecdison	1451935	BI	11515	5.8	4.4	1.0	33.3

Figure S3. HPLC chromatogram of H1 - β-ecdysone

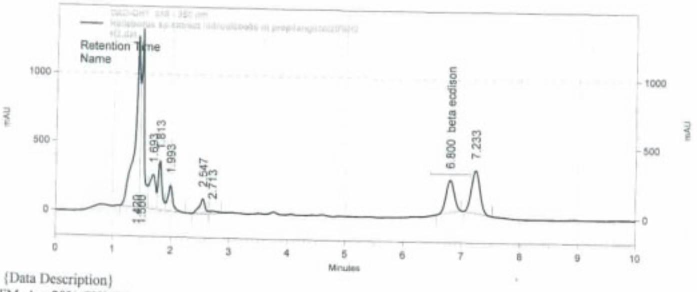

{Data Description}
FM. A = 20% CH3CN
Temperatura =55 C
Debit = 1,5 ml/min
Detectie = 248 nm.
Coloana: Inertsil ODS 3 250*4.6*5 (42)

DAD-CH1 248 - 350 nm Results

Pk #	Name	RT	Area
8	beta ecdison	6.800	8936115

DAD-CH1 248 - 350 nm Results

Pk #	Name	Height	IC	PLATES	K	Res	As	S/N
8	beta ecdison	923295	BI	11010	5.8	0.0	1.0	16.9

Figure S4. HPLC chromatogram of H2 - β-ecdysone
